# Value of Experiment in Preventive Medicine

**Published:** 1881-12

**Authors:** 


### POPULAR SCIENCE DEPARTMENT.

### THE VALUE OF EXPERIMENT IN PREVENTIVE MEDICINE.

AN ABSTRACT OF DR. JOHN SIMON'S ADDRESS BEFORE THE SECTION ON PUBLIC MEDICINE AT THE INTERNATIONAL MEDICAL CONGRESS.

The experiments which give us our teaching with regard to the causes of disease are of two sorts: on the one hand we have the carefully pre-arranged and comparatively few experiments which are done by us in our pathological laboratories, and for the most part on other animals than man; on the other hand we have the experiments which accident does for us, and, above all, the incalculably large amount of crude experiment which is popularly done by man on man under our present ordinary conditions of social life, and which gives us its results for our interpretation.

When I say that experiments of those two sorts are the sources from which we learn to know the causes of disease, I, of course, do not mean that the mental process by which an experiment becomes instructive to us is the same in regard of the two sorts of experiment. On the contrary, the etiological problem (so long as it is a problem) is approached in the two cases from two opposite points of view; and the dynamical continuity of relation, which we call cause and effect, is traced, in the one case, from the one pole, and in the other case, from the other pole of the relation. In the one case, starting with knowledge of our own deliberately-prepared *cause*, our question is, What will be its effect? In the other case, starting from a certain *effect* presented to us, our question is, What has been its cause? But in the second case, just as in the first, when the question is answered, when the problem is solved, when the relation of cause and effect has been made clear, we recognize that the conjuring-power which has brought us our new knowledge is the power of a *performed experiment.*

Let me illustrate my argument by showing you the two processes at work in identical provinces of subject-matter. What are the classical experiments to which we habitually refer when we think of guarding against the dangers of Asiatic cholera? On the one side there are the well-known *scientific* infection-experiments of Prof.

Thiersch, and others following him, performed on a certain number of mice. On the other hand there are the equally well-known *popular* experiments, which, during our two cholera epidemics of 1848–9 and 1853–4, were performed on half a million of human beings, dwelling in the southern districts of London, by certain commercial companies which supplied those districts with water. Both the professor and the companies gave us valuable experimental teaching as to the manner in which cholera is spread. I need not state at length the facts of those experiments, probably known to all here, but may rather justify my parallel by referring to an etiological question which will presently be discussed in our section.

It concerns the *causation of tubercle*—the most fatal by far of all the diseases to which the population of this country is subject. On that subject, for the last sixteen years, we have had a new era of knowledge. It was the great merit of a Frenchman, M. Villemin, that he, in 1865, first made us fully aware that tubercle is an infectious disease. He did this by certain *laboratory experiments* performed on other animals than man. He found that general and fatal tubercular infection of the animal was produced when he inoculated it subcutaneously with a little crude tubercular matter from the human subject. That first laboratory investigation of the subject has been followed most extensively by others; and the further experiments, while entirely confirming M. Villemin's discovery, have shown that subcutaneous inoculation is not the only mode by which the tubercular infection can be propagated. Dr. Tappeiner and others have shown that the same effect is produced on the animal if tubercular matter, (such as the sputa of phthisical patients) be diffused in spray in the air which the animal breathes; and Prof. Gerlach of Hanover showed twelve years ago, with regard to the bovine variety of tubercular disease (the perlsucht of the Germans), that its infection can be freely introduced through the stomach if bits of tubercular organs be given in the food, or if the healthy animal be fed with milk from the animal which has tubercle. That the communicability of tubercle from animal to animal is also being tested to an immense extent by *popular experiments* on the human subject, is what a moment's reflection will tell; and from that wide field of experiment I select one instance for illustration. I have every reason to believe that Prof. Gerlach's experiments on the communicability of tubercle by means of milk are very extensively parodied by commercial experiments on the human subject. I learn, on what I believe to be the highest authority in this country, that tubercle (in different degree) is a malady which abounds among our cows; and that so long as the cow continues to give milk, no particular scruple seems to prevent a distribution of that milk for popular use. To the persons who consume that milk an important question as to the causability of tubercle is put in an experimental form. Whether they will become infected with tubercle is a question which the individual consumers do not stand forward to answer for themselves like the animals of the laboratory experiments; but Dr. Creighton's lately published book, entitled, "Bovine Tuberculosis in Man," and a paper in which I am glad to say he brings under notice of our section the very remarkable series of facts on which he grounds that startling title seem to suggest a first installment of answer in accordance with Prof. Gerlach's experimental finding.

The two sorts of experiment—the scientific and the popular—differ, as I have noted, in this particular; that the popular experiment is almost always done on man; the scientific almost always on some other animal. It is true that many memorable cases are on record, where members of our profession have deliberately given up their own persons to be experimented on by themselves or others for the better settlement of some question as to a process of disease; have deliberately tried, for instance, whether, in this way or in that, they could infect themselves with the poison of plague or of cholera; and as regards one such case which is in my mind, I think it not unlikely that the illustrious life of John Hunter was shortened by the experiments which he did on himself with the ignoble poison of syphilis. There have been cases, too, where criminals have been allowed to purchase exemption from capital or other punishment at the cost of allowing some painful or dangerous experiment to be performed on themselves. And cases are not absolutely unknown where unconsenting human beings have been subjected to that sort of experiment. But waiving such exceptions, the rule is, as I have said, that scientific experiments relating to causes of disease are performed on some animal which common opinion estimates as of lower importance than man. Now, as between man and brute, I would not wish to draw any distinction which persons outside this room might find invidious; but, assuming for the moment that man and brute are of exactly equal value, I would submit that, when the life of either man or brute is to be made merely instrumental to the establishment of a scientific truth, the use of the life should be economical. Let me, in that point of view, invite you to compare, or rather to contrast with one another, those two sorts of experiment from which we have to get our knowledge of the causes of disease. The commercial experiments which illustrated the dangerousness of sewage-polluted water-supplies cost many thousands of human lives; the scientific experiments which with infinitely more exactitude justified a presumption of dangerousness, cost the lives of a few dozen mice. So, again, with experiments as to the causation of tubercle:—judging from the information which I quoted to you, I should suppose that the human beings whose milk supply on any given day includes milk from tubercular cows, might be counted, in this country, in tens of thousands; but the scientific experiments which justify us in declaring such milk supply to be highly dangerous to those who receive it were conclusive when they amounted to half a dozen. So far, then, as regards the mere getting of experimental knowledge, we must not, with a view to economy of life, be referred to popular, rather than scientific experiment. And in the same point of view, it perhaps also deserves consideration that the popular experiments, though done on so large a scale, very often have in them sources of ambiguity which lessen their usefulness for teaching.

The invaluable studies of M. Pasteur, beginning in the facts of fermentation and putrefaction, and thence extending to the facts of infectious disease in the animal body, where M. Chauveau's demonstration of the particulate nature of certain contagia came to assist them—they, I say, partly in themselves, and partly in respect of kindred labors which they have excited others to undertake, have introduced us to a new world of strange knowledge. We have learnt, as regards those diseases of the animal body which are due to various kinds of external cause, that probably all the most largely fatal of them (impossible yet to say how many), represent but one single kind of cause, and respectively depend on invasions of the animal body by some rapidly self-multiplying form of alien life. This doctrine, which scientific experiment initiated, has, for the last dozen years, been extending and confirming itself by further experiment. As soon as the doctrine began to seem probable, science saw that, should it prove true, it must have the most important corollaries. If the cause of an infecting human disease is a self-multiplying germ from the outside world, the habits of that living enemy of ours can be studied in its outside relations. It becomes an object of common natural history, it has biological affinities and analogies. We can cultivate it in test-tubes in our laboratories, as the gardener would cultivate a rose or an apple, and we can see what agrees and what disagrees with its life. And then, as the next and immeasurably most important stage, where nothing but experiment on the living body will help us, we can try whether perhaps any of our modifications of its life have made it of weaker power in relation to the living bodies which it invades, or whether, through our more intimate knowledge of its vital affinities, we can artificially give to bodies which it would invade, a partial or complete protection against it. Such, at first blush, were the obvious possibilities of research which the new doctrine of infectious disease suggested to the mind of the pathologist; and never since the profession of medicine has existed, had a field of such promise been before it. The promise has not been belied. A host of diseases has been worked at in such lines as I just now indicated, and with many of them important progress has been made.

It would be impossible for me even to name a twentieth part of the investigations which have been more or less successful. As regards some which have most struck me, I pass with but a word Dr. Klein's investigation of the pneumo-enteritis of swine; Prof. Cohn's and Dr. Koch's and Dr. Buchner's respective contributions to the natural history of the anthrax bacillus; Dr. Bollinger's recognition of the microphytic origin of an important cancroid disease of horned cattle, with Dr. Johne's illustration of the inoculability of this disease; the research by Drs. Klebs and Tommasi-Crudeli into the intimate cause of marsh-malaria; and, not least, the demonstration (as it appears to be) which Dr. Grawitz has recently published, that some of the commonest and most innocent of our domestic microphytes can be changed by artificial culture into agents of deadly infectiveness. I pass these and others, in order that I may more particularly speak of some which have already shown themselves practically useful; for in respect of some of them the time has already come when abstract scientific knowledge is passing into preventive and curative knowledge.

First, and not in a spirit of national partiality, I will mention the application which M. Pasteur's doctrine has received at the hands of Mr. Lister, with regard to the antiseptic treatment of wounds: an application which, enforced and illustrated at every turn by Mr. Lister's own eminent skill as an experimentalist, has been confirmation as well as application of the parent doctrine; and the beneficent uses of which, in giving comparative safety to the most formidable surgical operations, and in immensely facilitating recovery from the most dangerous forms of local injury, are recognized—I think I may say, by the grateful common consent of our profession in all countries, to be among the highest triumphs of preventive medicine.

Secondly, out of the experimental studies of anthrax—chiefly out of those of Dr. Sanderson and Mr. Duguid in this country, and those of Dr. Buchner, in Germany, and M. Toussaint, in France, has grown a knowledge of various ways in which the contagion of that dreadful disease can be so mitigated that an animal inoculated with it, instead of incurring almost certain death, shall have no serious illness; and the further knowledge has been gained that the animal submitted to that artificial procedure is thereby more or less secured against subsequent liability to the disease. In other words, with regard to that disease, an infliction which sometimes spreads to man from his domestic animals, and one which in some parts of Europe is of serious consequence to agricultural interests, as well as to animal life, the later experimenters—of whom I may particularly name M. Toussaint and our countryman, Prof. Greenfield—seem to have given to the animal kingdom, and to the farmers, the same sort of boon as that which Jenner gave to mankind when he taught men the use of vaccination. Quite recently, our great leader, M. Pasteur, seems to have made, by new experiments, still further progress in the mitigation of anthrax.

Thirdly, a similar discovery has been made by M. Pasteur, with reference to the contagium of a very fatal poultry disease, known by the name of fowl's cholera; he has learned to mitigate that contagium to a degree, in which, if fowls be inoculated with it, they will suffer no serious ailment; and he has found that fowls so inoculated (or, as he, in honor of Jenner, would say, "fowls so vaccinated") are proof against future attacks of the disease.

Fourthly, Prof. Semmer, of Dorpat, through experiments done under his direction by Dr. Krajewski, has made a similar discovery in regard of the infection of septicæmia; has found, namely, that by treatment like that with which M. Toussaint mitigates the contagium of splenic fever, he can bring the most virulent septic contagium into a state in which it shall be mild enough to serve for harmless inoculations; which inoculations, when performed, shall be protective against future infections.

Finally, in a different direction of experimental work, let me name the recent and most admirable research which Dr. Schuller, of Greifswald, has made nominally in respect of certain surgical affections of joints, but in reality extending to the inmost pathology and therapeutics of all tubercular and scrofulous affections. A knowledge of the fatal infectiveness of crude tubercular matter had been given (as I before said) by Villemin and those who followed him; and Prof. Klebs, four years ago, declared the infective quality to be due to the presence of a microphyte (micrococcus), which he had succeeded in separating from the rest of the matter by successive acts of cultivation in fluids of inorganic origin. Dr. Schuller solidly settles and widely extends that teaching. According to his apparently quite unquestionable observations and experiments, the micrococcus which characterizes tubercle, characterizes also certain affections popularly called, "scrofulous"—namely, "scrofulous" synovial membrane, "scrofulous" lymph-glands, and lupus: so that these diseases may be defined as essentially tubercular, and that inoculation with matter from any of them, or with a cultivation fluid in which the micrococcus from any of them has been cultivated, will infect with general tuberculosis. The rapid multiplication of the tubercle miscrococcus in the blood and tissues of any inoculated animal can be verified both by microscopical observation, and by inoculative experiment; and an extremely interesting part of the research, in explanation of certain of our human joint-diseases, is the demonstration that if in the inoculated animal a joint is experimentally injured, that joint at once becomes a place of preferential resort to the micrococcus which is multiplying in the blood, and becomes consequently a special or exclusive seat of characteristic tubercular changes. Even thus far the practical interest of Dr. Schuller's book is such as it would not be easy to overstate, but still greater interest attaches to the last chapter of the book, in which, confidently resting on the pathological facts which I have quoted, he makes proposals for the treatment of tubercle on the basis of its microphytic origin, and shows the successful result of such treatment as he has hitherto tried, from that basis, on animals artificially infected by him.

I venture to say that in the records of human industry it would be impossible to point to work of more promise to the world than these various contributions to the knowledge of disease, and of its cure and prevention; and they are contributions which from the nature of the case have come, and could only have come, from the performance of experiments on living animals.

Dr. Simon here refers to the Act of Parliament, against vivisection, which, at present, acts as a practically absolute bar against the performance of scientific experiments on animals in Great Britain. After pointing out the absurdity of the law, making the Secretary of State the judge and regulator of the details of experimental procedure, he continues:—

Consider for a moment what this means in regard of the members of our profession whom it affects. Contrast with it the almost unbounded trust with which the world, from time immemorial, has regarded the character of our profession. Consider the relation of inmost confidence in which members of our profession in every corner of the kingdom are admitted to share in the sanctities and tendernesses of domestic life. Consider our immense daily responsibilities of human life and death. Consider that there is not a member of our profession to whom the law does not allow discretion that, in certain difficulties of childbirth, he shall judge whether he will kill the child to save the mother. And in contrast with all this, is it to be seriously maintained that society cannot trust us with dogs and cats? that our foremost workers—(for it is essentially they who are affected)—cannot be trusted to behave honestly towards their brute fellow-creatures, unless they be regulated and inspected under a special law in much the same prevenient spirit as if they were prostitutes under the Contagious Diseases Act?

I have reason to believe that, if that Act continues on the statute-book, one of two results will follow. Experiments, indispensably necessary for the growth of medical science in relation to the cure and prevention of disease, will cease, or almost cease, to be done in this country; or, as the alternative to this, persons who desire to advance the science of their profession, will be tempted to clandestinely ignore the law and run their chance, if the worst comes to the worst, of having to try conclusions with the common informer.

Let me illustrate this by two personal references: I have already mentioned Prof. Lister as an experimenter, whose name is now classical wherever science has reached, and whose work has been of signal advantage to mankind. Last autumn Mr. Lister wished to do some experiments in extension of the particular branch of knowledge with which his name is identified, and at a point which he considered of extreme importance in surgical pathology. He found he must either abandon his investigation or must conduct it in a foreign country, and in his zeal for science he chose the latter course. His experiments (which had to be on large animals) were done at the Veterinary College of Toulouse; and in stating this fact in a letter, from which I quote, Mr. Lister added that "even with reference to small animals, the working of the Act is so vexatious as to be practically prohibitory of experiments by a private practitioner like myself, unless he chooses to incur the risk of transgressing the law." A second name which I have mentioned is that of Prof. Greenfield, who has so highly distinguished himself in developing, by means of experiments, the preventive medicine of splenic fever. Dr. Greenfield, in order to perform his inoculation experiments, had of course to become a license-holder under the Act; and his experience of the hindrances which attach to that position is expressed to me in the following terms: "It is my deliberate conviction, as a result of my experience, that these hindrances and obstacles are so numerous and so great as to constitute a most serious bar to the investigation of disease, and even of such remedial measures as would by common consent be for the direct benefit of the animals experimented upon. When to this is added all the annoyance and opprobrium which are the lot of investigators, it is to be wondered at that any one should submit to be licensed." Dr. Greenfield's experimental operations consisted only in inoculating the virus of animal diseases, and he says: "I have not been engaged in other investigations for the simple reason that, with the present restrictions and the difficulty in obtaining a license, I regard it as almost hopeless to attempt any useful work of the kind in this country."

As I feel sure that the Act must at no distant time be reconsidered by the Legislature, and as I also very strongly feel that, quite apart from any question of legal enactments, there is the question of moral right or wrong to be considered in the matter, I would beg you to allow me to make my own public confession of faith (from which I dare say yours will not much differ) in that extremely important matter of controversy.

The question being whether medical science can rightly use living animals as subjects for experiments which may be painful, and even, in exceptional cases, very painful to them, the answer may be sought in either of two directions: 1. What says the voice of the experimenter's own conscience? and 2. What says the standard of common contemporary conduct in analogous cases?

As regards the first, if I may take the liberty of expressing my own feeling, I would say this. I do not in any degree regard it as a matter of indifference that, in certain cases, by my own hand or by that of some one acting for me, I must inflict death or pain on any living thing, I, on the contrary, think of it with true compunction; but I think of it as good or bad according to the end which it subserves. Where I see my way to acquire, at that painful cost, the kind of exact knowledge which, either in itself or in contribution to our common stock, will promote the cure or prevention of disease in the race to which the animal belongs, or in the animal kingdom generally, or (above all) in the race of man, I no more flinch from what then seems to be a professional duty, though a painful one, than I would, in the days before chloroform, have shrunk from the cries of a child whom I had to cut for stone. If, in the case of the latter sort, the surgeon nerves himself to his work by the conviction of an indispensable usefulness in what he has to do, so does the pathologist in his, and surely in a much larger sense. The agitated parent of the child might sometimes be tempted to say: " Forbear giving this cruel pain; let the poor little sufferer die;" but the surgeon's reply would have comforted her. And so with the physiological experimenter: except that he, instead of looking at one individual life to be saved, is looking at a race or at many races, and reflects how, in respect of some grevious physical misery, the whole of them, in all their multitudinous successions, may be redeemed through the suffering of the few. This is my personal view of the abstract right or wrong in the question. I state it because, in matters of right and wrong, no man ought to shelter himself behind authority. I believe I may add that if it, or something very like it, had not for centuries been the general view of the medical profession, our professional knowledge would probably be standing in this present age about where it stood in the days of the Plantagenets. Of Harvey and Hunter and Bell, we well know that such was the view on which they acted in rendering their immortal services to mankind; and I am not aware that any man, whose opinion really counts in matters of medical science, would express any material dissent from it.

The second standard to which I referred was that of the common conduct of men in *analogous* cases. I pointedly say "analogous," rather than "similar," because common life does not in fact give cases which, properly speaking, are "similar," to ours. But what, I ask is the common *principle* of behavior of civilized man towards the so-called lower animals? He in every respect subordinates their lives to his own. If he thinks he can get an advantage to himself by killing or painfully mutilating an animal, he does so with apparently no hesitation. See, for instance, the sexual mutilations which are inflicted on all but a small minority of most kinds of domestic animals, and, as regards some kinds, on many of the females.

I protest against any man's applying to this extremely important question a purely arbitrary standard of right or wrong. Those who pronounce judgment on their neighbors must be prepared to state the principle on which they judge. "Compound for sins you are inclined to, by damning those you have no mind to," is the Pharisee's easy-going formula. Where would life be if that were generally accepted? Suppose a *genus* of action; let men draw an arbitrary line across it —a line prescribed by no better rule than that which governed the lady's dislike to Dr. Fell; let them affix a nickname of praise to all on one side of the line, and a nickname of dispraise to all on the other: truly we should thus have the readiest of royal roads to unlimited mutual persecution.

And I protest against a standard of right and wrong being fixed for us on grounds which are merely sentimental. In certain circles of society, at the present ti ne, æsthetics count for all and all; and an emotion against what they are pleased to call "vivisection" answers their purpose of the moment as well as any other little emotion. With such sections of society, our profession cannot seriously argue. We have to think of usefulness to man. And to us, according to our standard of right and wrong, perhaps those lackadaisical æsthetics may seem but a feeble form of sensuality.

Of the mere screamers and agitation-mongers who, happy in their hysterics or their hire, go about day by day calumniating our profession and trying to stir up against it the prejudice and passions of the ignorant, I have only to express my contempt.

I regret to have had to speak at so much length of the heavy cloud which at present hangs over the study of scientific medicine in England, and which, in my opinion, is likely to be of specially disastrous effect on the progress of preventive medicine. As a very old public servant in that cause, I should indeed grieve to see it brought to a stand-still for want of the scientific nurture which, in truth, is its very basis of life; and, speaking publicly of the danger on this occasion, I have hoped that the occasion may give importance to what I say.

And now, gentlemen, from contemplating that cloud, which happily is but local, and which perhaps may be but temporary, I gladly turn to skies which have no cloud. If there exist in the social organism any function whatsoever for which development and eventual triumph may be foretold, surely it is that of State Medicine. Of the two great factors concerned in it—the two strong powers which within our own time have converged to make it the reality which it is—the growth of science on the one hand, and the growing stress of common humanity on the other, neither one is likely to fail. Of our science it is needless to say that it will grow. To the science of nature indeed is allotted that one incomparable human day which knows no sunset. In the pure light of its ever-present daybreak, individual workers will pass away, generations will change, but the studies of Nature, and, above all, the gathering of such knowledge as can lessen man's physical difficulties and sufferings, will surely grow from age to age, and, as on Proserpina's sacred tree, one golden fruit will follow another: "simili frondescet virga metallo." And no less also in the other direction, the auguries are wholly for our cause. Popular education is gradually making its way, and it will grow to be a force on our side. Masses of mankind that now have to be humbly pleaded for by others, will then be strong to speak for themselves. Physical interests, now but little understood, will then be within grasp of all men's apprehension. Not only will health be recognized at its true value, and its elementary requirements be regarded, but also the frauds and villainies which are now committed against it will have become intelligible to the common mind; and the workman of the future will strike against being cheated in health as he would now strike against being cheated in wages. As such times come to the world, the science and the profession which care for man as man will get to be better appreciated than now. And in proportion as an educated people grows to become Body-Politic, State Medicine will be seen to represent the true ideal of Government-action which sets its standard of success in the "greatest happiness of the greatest number."

